# Supplementary material for: Sandwich Enzyme-Linked Immunosorbent Assay for Quantification of Callose
Source: Methods Protoc. 2022 Jun 26;5(4):54. doi: 10.3390/mps5040054 (PMC9326611; doi:10.3390/mps5040054)
Supplement: Supplementary file 1 [file mps-05-00054-s001.zip › mps-1709713-supplementary.pdf]

Supplementary Table S1: Analysis of callose concentration in the leaves pseudostems and corms of banana plants inoculated and non-inoculated (control) with *Xcm* (Independent sample t-test, $\alpha \leq 0.05$).

Banana genotype	Banana tissue	Treatment	Mean callose concentration ± SD in µg/mL (LE)	N	Shapiro-Wilk test (ρ)	F-test (ρ)	t-test		
							t	df	ρ
Mbwazirume	Leaves	Control	41.72 ± 5.64	8	0.1393	0.08402	4.9520	12	0.0003353
		Xcm-inoculated	64.91 ± 11.66	6	0.5634				
	Pseudostems	Control	1719.15 ± 341.95	9	0.3605	0.5169	2.0954	17	0.05142
		Xcm-inoculated	2097.35 ± 433.08	10	0.139				
	Corms	Control	41440.47 ± 5745.31	7	0.01729	0.1172	7.2059	13	0.000006888
		Xcm-inoculated	82981.50 ± 14217.08	8	0.5637				
Musa balbisiana	Leaves	Control	34.18 ± 4.41	6	0.8215	0.06783	6.2617	14	0.00002085
		Xcm-inoculated	62.96 ± 10.60	10	0.5359				
	Pseudostems	Control	1820.05 ± 428.92	7	0.2492	0.6476	2.0771	13	0.05818
		Xcm-inoculated	2337.56 ± 522.21	8	0.4627				
	Corms	Control	32758.00 ± 4917.78	7	0.3807	0.1956	4.9106	14	0.0002297
		Xcm-inoculated	50612.83 ± 8541.61	9	0.9799				
